# Bedside echocardiography in critically ill patients

**DOI:** 10.1590/S1679-45082015MD3271

**Published:** 2015

**Authors:** Eduardo Casaroto, Tatiana Mohovic, Lilian Moreira Pinto, Tais Rodrigues de Lara

**Affiliations:** 1 Hospital Israelita Albert Einstein, São Paulo, SP, Brazil.

**Keywords:** Echocardiography, Intensive care, Hemodynamics, Cardiac output, Shock, Ventricular function

## Abstract

The echocardiography has become a vital tool in the diagnosis of critically ill patients. The use of echocardiography by intensivists has been increasing since the 1990’s. This tool has become a common procedure for the cardiovascular assessment of critically ill patients, especially because it is non-invasive and can be applied in fast and guided manner at the bedside. Physicians with basic training in echocardiography, both from intensive care unit or emergency department, can assess the left ventricle function properly with good accuracy compared with assessment made by cardiologists. The change of treatment approach based on echocardiographic findings is commonly seen after examination of unstable patient. This brief review focuses on growing importance of echocardiography as an useful tool for management of critically ill patients in the intensive care setting along with the cardiac output assessment using this resource.

## INTRODUCTION

The echocardiography has evolved significantly over the last years and, since 2001, this technique was included in clinical practice for cardiac output assessment.^(1)^ This is non-invasive technique with a variety of benefits, compared with other cardiovascular assessment techniques, in terms of safety, low cost, wide availability, absence of radiation exposure and need of using contrast. In addition, it is an portable device, causes minimal discomfort to the patient, shows immediate results and does not require displacement of the patient to the imaging department.^(1-4)^ This device can assess the heart structurally, functionally and hemodynamically,^(4,5)^ and its importance have been recognized by several scientific society including the British Society of Echocardiography, the American Society of Echocardiography and the World Interactive Network Focused on Critical Ultrasound.^(6)^ The major indications for bedside echocardiography are described in chart 1.

**Chart 1:** Major indications to hemodynamic echocardiography in the intensive care unit(2,3,6-8)

Hypotension/hemodynamic instability of unknown etiology
Fluid responsiveness assessment
Evaluation of severe dysfunction of right ventricle
Identification of pericardial effusion/cardiac tamponade
Respiratory failure or hypoxemia of unknown etiology
Pulmonary embolism
Complications after cardiothoracic surgery

Echocardiogram is a tool that support diagnosis, monitoring, management and clinical progress of critically-ill patients,^(7,8)^ in addition it works as therapeutic interventions.^(2,3)^ A number of non-cardiology specialists as anesthesiologist, intensivists and emergency physician have used brief approaches directed to specific findings,^(2,3,8)^ and they achieve reasonable accuracy including at other environments, not only in intensive therapy settings.^(9)^ This exam can be called “echohemodynamic”, “point of care” echo, among other.^(8)^ The examiner should be able to evaluate left and right ventricular function, volemic status and pericardial space as basic requirements.^(5)^ A guided-exam for specific problem has duration significantly shorter than complete echocardiogram – about 6 minutes or shorter.^(5)^ The intensivist compared with other specialists need more complex information, such as ventricular filling pressure, contractility and cardiac output.^(7)^ The fluid responsiveness can also be evaluated, the need of inotropic and/or vasopressor agents, and evaluate mechanical ventilation impact.^(7,10)^ Other assessments, such as changes in vascular function and estimative of pulmonary capillary wedge pressure, require more advanced training.^(5)^

Basic competencies in echocardiographs for intensivists according to Cholley et al.^(11)^ include the ability to differentiate the systolic function of left ventricle (LV) in normal, moderate or severe dysfunction, right ventricular dilatation, collapse or dilatation of inferior vena cava, and pericardial effusion.^(2)^

When the main causes of interpretation mistakes in bedside echo are analyzed, the biggest mistake is related to non-recognition of depressed function of LV, which is more common than interpret a normal function of LV as abnormal.^(9)^ In general, there is a tendency to overestimate the function of LV.^(9)^ In addition, less trained individual can fail to recognize other important causes of hemodynamic compromising, such as acute *cor pulmonale* and acute valvular abnormalities.^(9)^

It is important to emphasize that complete echocardiogram carried out by the adequate professional can identify standard windows and adequate cardiac and valve function, as well as structural data and a number of other information.^(8)^ Unfortunately, the possibility of performing a echocardiography and get immediate interpretation by a cardiologist is not always available in the intensive care unit.^(3,9)^

To become an expert in echocardiography requires adequate training to guarantee quality and reliability for the exam. Adequate training avoid risks of poor interpretation, but it is still a challenge for most of intensivists.^(3,8,10)^ A ultrasonography exam requires anatomical knowledge by the examiner for correct assessment of the patient, in addition the examiner need to have knowledge on physics for adequate operation of the device.^(4)^

The intensive therapy environment entails a number of difficulties to perform the exam: suboptimal lightning conditions, drainages, edema, rapid oscillations in hemodynamic status, ventilations, and difficulties related to patients.^(4)^ Chart 2 shows advantages and disadvantages in the use of hemodynamic echocardiography in the intensive therapy settings.

**Chart 2:** Advantages and disadvantages of echocardiography in intensive therapy settings(4)

Advantages	Disadvantages
Information previously obtained to invasive monitoring	Intermittent measures
There is no need of other professional, expect the physician who do the exam	No acquisition of all echocardiographic windows
Real-time data	Low offering of training programs
Safety and portable	

Most limiting factors of the echocardiography use are its intermittent character and its operating system dependent.^(10)^ Additionally there is the low offering of practical training programs in hemodynamic echocardiography, especially for intensivists.^(5)^ Chart 3 shows other examples.

**Chart 3:** Limitations and challenges of transthoracic echocardiogram(4)

Multiple windows frequently needed
Repositioning of the patient is usually necessary
Particular characteristics of each patient can interfere in image acquisitions
Interference with monitoring devices

There are new and cheaper battery-powered devices that are becoming more and more available, and constitute an excellent tool to be used by the intensivists.^(3,4)^ Some experts also suggest that echocardiogram should be used as an adjunctive tool for physical exam.

All intensivists should be able to perform at least one brief examination in a shock situation with unknown etiology. Based on all information we exposed above, to know how to perform an echocardiography should be considered a prerequisite for intensivists.^(3,5)^

## CONCLUSION

Hemodynamic assessment was always one of the basics for intensive therapy and for unstable patients at this service. The use of ultrasonography is already well consolidated. It has good accuracy, is a non-invasive, do not expose the patient to radiation and can be performed at bedside. Ultrasonography is no longer performed only by radiologists. Anesthesiologists, intensivists and emergency physicians also use this procedure in daily clinical practice. Today is becoming common to find a non-cardiology physician performing an echocardiography focused on specific findings, especially for diagnosis and management of critically unstable patients. To obtain adequate images, and knowledge about limitations and fails of echocardiography is key to achieve an adequate performance with the equipment in intensive care units. Although one of the limiting factors to improve this technique application is still the lack of adequate training programs, in the near future training in echocardiography for intensivits and emergency physicians will be probably part of their education curriculum.
