# Supplementary material for: Bedside echocardiography in critically ill patients
Source: Einstein (Sao Paulo). 2015 Oct-Dec;13(4):644–6. doi: 10.1590/S1679-45082015MD3271 (PMC4878647; doi:10.1590/S1679-45082015MD3271)
Supplement: Translation

# Translation: Ecocardiografia à beira leito em pacientes graves

**Authors:** Eduardo Casaroto, Tatiana Mohovic, Lilian Moreira Pinto, Tais Rodrigues de Lara

**Affiliations:** 1 Hospital Israelita Albert Einstein, São Paulo, SP, Brasil.

**Keywords:** Ecocardiografia, Terapia intensiva, Hemodinâmica, Débito cardíaco, Choque, Função ventricular

## Abstract

A ecocardiografia tem se tornado uma ferramenta fundamental no atendimento ao paciente grave. Desde os anos 1990, o uso do ecocardiograma por intensivistas vem aumentando progressivamente. É cada vez mais frequente encontrarmos o ecocardiograma como parte do arsenal diagnóstico na avaliação cardiovascular dos pacientes graves, visto se tratar de uma ferramenta não invasiva, que pode ser utilizada à beira do leito de maneira rápida e direcionada. Seja na unidade de terapia intensiva ou no departamento de emergência, médicos com mínimo treinamento em ecocardiografia são capazes de avaliar a função do ventrículo esquerdo de maneira adequada e com bom nível de concordância com a interpretação dos cardiologistas. Não é incomum constatarmos alteração de conduta baseada nos achados ecocardiográficos em avaliação realizada na abordagem inicial ao paciente instável. Nessa breve revisão, o foco está voltado à crescente importância ao ecocardiograma como ferramenta extremamente útil na abordagem aos pacientes graves no ambiente da terapia intensiva, aliado à avaliação do débito cardíaco, utilizando esse recurso.

## INTRODUÇÃO

A ecocardiografia tem evoluído significativamente e, desde 2001, vem sendo incorporada à prática clínica como método de avaliação do débito cardíaco.^(1)^ Consiste em um método não invasivo, com uma série de benefícios sobre outros métodos de avaliação cardiovascular: segurança, custo relativamente baixo, ampla disponibilidade, ausência de radiação ou uso de contraste, mínimo desconforto ao paciente e resultados imediatos, além de ser portátil e não exigir deslocamento do paciente ao setor de imagem.^(1-4)^ É capaz de avaliar o coração do ponto de vista estrutural, funcional e hemodinâmico,^(4,5)^ e sua importância tem sido reconhecida por várias sociedades, incluindo a *British Society of Echocardiography*, a *American Society of Echocardiography* e a *World Interactive Network Focused on Critical Ultrasound.* ^(6)^ No quadro 1, encontramos as principais indicações para realização do ecocardiograma à beira do leito.

**Quadro 1:** Principais indicações para o emprego da ecocardiografia hemodinâmica na terapia intensiva(2,3,6-8)

Hipotensão/instabilidade hemodinâmica de etiologia indefinida
Avaliação de responsividade a fluidos e volemia
Avaliação de disfunção grave de ventrículo direito
Identificação de derrame pericárdico/tamponamento
Insuficiência respiratória ou hipoxemia de etiologia indefinida
Embolia pulmonar
Complicações após cirurgia cardiotorácica

O ecocardiograma é uma ferramenta que possibilita avaliação diagnóstica, monitorização, evolução e manejo dos pacientes graves,^(7,8)^ servindo, inclusive, como guia às intervenções terapêuticas.^(2,3)^ Desse modo, vários especialistas não cardiologistas, como anestesistas, intensivistas e emergencistas, o tem utilizado com abordagens sucintas, direcionadas a achados específicos,^(2,3,8)^ alcançando uma acurácia razoável inclusive em ambientes outros, que não terapia intensiva.^(9)^ Essa modalidade de exame pode ser chamada de “eco-hemodinâmico”, “ecofocado”, “point of care” eco, entre outras denominações,^(8)^ e o examinador deve ser capaz de avaliar função ventricular esquerda e direita, estado volêmico e espaço pericárdico, como requisitos básicos.^(5)^ Um exame dirigido a um questionamento específico tem duração significativamente menor que um ecocardiograma completo − cerca de 6 minutos ou menos.^(5)^ O intensivista, comparado a outros especialistas, necessita informações mais complexas, como pressões de enchimento, contratilidade e débito cardíaco.^(7)^ Também é possível avaliar a responsividade a fluidos, a necessidade de inotrópico e/ou vasopressores, e, ainda, inferir o impacto da ventilação mecânica.^(7,10)^ Avaliações outras, como alterações da função valvular e estimativa de pressão de oclusão do capilar pulmonar, necessitam de um treinamento mais avançado.^(5)^

As competências básicas em ecocardiografia para intensivistas segundo Cholley et al.^(11)^ envolvem a capacidade de diferenciar a função sistólica do ventrículo esquerdo (VE) em normal, disfunção moderada ou grave, dilatação ventricular direita, colapso ou dilatação da veia cava inferior, e derrame pericárdico.^(2)^

Quando se analisam as principais causas de erro na interpretação do eco à beira do leito, nota-se que o maior equívoco se relaciona à falha em reconhecer uma função deprimida de VE, mais comum do que interpretar uma função normal de VE como anormal.^(9)^ No geral, a tendência é superestimar a função do VE.^(9)^ Além disso, um indivíduo pouco treinado pode falhar em reconhecer outras importantes causas de comprometimento hemodinâmico, como *cor pulmonale* agudo e anormalidades valvares agudas.^(9)^

Ressalta-se que o ecocardiograma completo realizado pelo profissional adequadamente habilitado é capaz de identificar as janelas padrão e a adequada função cardíaca e valvular, bem como dados estruturais e uma série de outras informações.^(8)^ Infelizmente, nem sempre temos a possibilidade de realização do ecocardiograma e a interpretação do cardiologista prontamente disponível na terapia intensiva.^(3,9)^

A *expertise* em ecocardiografia requer treinamento adequado para garantir qualidade e confiabilidade ao exame, além de evitar riscos de má interpretação e ainda representa um desafio para boa parte dos intensivistas.^(3,8,10)^ Realizar um exame de ultrassonografia requer conhecimento anatômico, para a correta avaliação do paciente, e conhecimentos de física, para o adequado manejo do aparelho.^(4)^

O próprio ambiente da terapia intensiva impõe uma série de dificuldades à execução do exame: condições de luminosidade subótimas, drenos, edema, rápidas oscilações no *status* hemodinâmico e ventilatório, além de dificuldades inerentes ao próprio paciente.^(4)^ O quadro 2 demonstra algumas vantagens e desvantagens no uso da ecocardiografia hemodinâmica na terapia intensiva.

**Quadro 2:** Vantagens e desvantagens da ecocardiografia na terapia intensiva(4)

Vantagens	Desvantagens
Informações obtidas previamente à monitorização invasiva	Medidas intermitentes
Não há necessidade de outros profissionais, além do médico que realiza o exame	Não obtenção de todas as janelas ecocardiográficas
Informação em tempo real	Baixa oferta de programas de treinamento
Seguro e portátil	

Os fatores mais limitantes no uso da ecocardiografia são seu caráter intermitente e o fato de ser operador-dependente.^(10)^ Soma-se a isso a baixa oferta de programas de treinamento prático em ecocardiografia hemodinâmica, especialmente destinados ao intensivista.^(5)^ O quadro 3 demonstra outros exemplos.

**Quadro 3:** Limitações e desafios com ecocardiograma transtorácico(4)

Múltiplas janelas frequentemente necessárias
Reposicionamento do paciente é usualmente necessário
Características próprias de cada paciente podem interferir na aquisição das imagens
Interferência com dispositivos de monitorização

Novos aparelhos portáteis, que funcionam com bateria e de custo menos elevado encontram-se cada vez mais disponíveis e representam uma ótima ferramenta para uso pelo intensivista.^(3,4)^ Há quem sugira, inclusive, que o ecocardiograma possa ser utilizado como uma extensão ao exame físico.

Todo intensivista deveria ser capaz de realizar ao menos um exame sumário em uma situação de choque com etiologia indeterminada, e até mesmo ser considerado um pré-requisito para sua atuação diante de tudo o que foi exposto logo acima.^(3,5)^

## CONCLUSÃO

A avaliação hemodinâmica sempre foi um dos cernes da terapia intensiva e seus pacientes instáveis. O uso da ultrassonografia já é bem consolidado: boa acurácia, não invasivo, não expõe o paciente à radiação e pode ser realizado no próprio leito. Seu uso já não é mais exclusivo dos radiologistas. Anestesistas, emergencistas e intensivistas utilizam o método na prática clínica diária. É cada vez mais comum encontrarmos um médico não cardiologista realizando ecocardiograma focado em achados específicos, voltado principalmente ao diagnóstico e ao manejo de pacientes gravemente instáveis. A obtenção de imagens adequadas, e o conhecimento das limitações e falhas do método é a chave para uma adequada *performance* com o equipamento na terapia intensiva. Apesar de um dos fatores limitantes à técnica ainda ser a falta de programas de treinamento adequados, muito em breve o treinamento em ecocardiografia dirigido para emergencista/intensivista provavelmente fará parte de sua formação.
